# Dr. Charles Auringer

**Published:** 1914-06

**Authors:** 


Dr. Charles Auringer, Syracuse Eclectic (1850-1857) 1855, died at his home in Detroit, April 15, aged 87. For more than 30 years, he was associated with the Michigan Mutual Life Insurance Co.

